# Fat Residue and Use-Wear Found on Acheulian Biface and Scraper Associated with Butchered Elephant Remains at the Site of Revadim, Israel

**DOI:** 10.1371/journal.pone.0118572

**Published:** 2015-03-18

**Authors:** Natalya Solodenko, Andrea Zupancich, Stella Nunziante Cesaro, Ofer Marder, Cristina Lemorini, Ran Barkai

**Affiliations:** 1 The Jacob M. Alkow Department of Archaeology and Ancient Near Eastern Cultures, Tel Aviv University, Ramat Aviv, Tel Aviv, Israel; 2 Scientific Methodologies Applied to Cultural Heritage (SMATCH), ISMN-CNR c\o Dept. of Chemistry, “Sapienza” Università di Roma, Rome, Italy; 3 The Department of Bible, Archaeology and Ancient Near Eastern Studies, Ben-Gurion University of the Negev, Beer-Sheva, Israel; 4 Department of Classics, “La Sapienza” University of Rome, Rome, Italy; University of Oxford, UNITED KINGDOM

## Abstract

The archaeological record indicates that elephants must have played a significant role in early human diet and culture during Palaeolithic times in the Old World. However, the nature of interactions between early humans and elephants is still under discussion. Elephant remains are found in Palaeolithic sites, both open-air and cave sites, in Europe, Asia, the Levant, and Africa. In some cases elephant and mammoth remains indicate evidence for butchering and marrow extraction performed by humans. Revadim Quarry (Israel) is a Late Acheulian site where elephant remains were found in association with characteristic Lower Palaeolithic flint tools. In this paper we present results regarding the use of Palaeolithic tools in processing animal carcasses and rare identification of fat residue preserved on Lower Palaeolithic tools. Our results shed new light on the use of Palaeolithic stone tools and provide, for the first time, direct evidence (residue) of animal exploitation through the use of an Acheulian biface and a scraper. The association of an elephant rib bearing cut marks with these tools may reinforce the view suggesting the use of Palaeolithic stone tools in the consumption of large game.

## Introduction

The most geographically wide spread and long-term cultural complex associated with the Lower Palaeolithic period in the Old World is the Acheulian. It is roughly dated between ca. 1.8-0.2 million years BP and commonly attributed to the hominin species *Homo erectus (senso lato)* [1, 2].

The Acheulian lithic technology is characterized by the production and use of flakes and flake tools, however the hallmark of Acheulian technology is known as the biface (also known as Handaxe and/or Large Cutting Tool [3]), a relatively large tool shaped on both faces providing evidence for manual dexterity, symmetry and prolonged use-life cycles [4, 5, 6, 7, 8, 9]. Other large tools such as choppers, cleavers and picks are also common in Lower Palaeolithic Acheulian sites world-wide, however bifaces represent a *fossil directeur* for the Acheulian cultural complex regardless of the fact that modern research have strongly indicated the variability and complexity of Acheulian lithic technology. Acheulian sites with few or even without bifaces are becoming integrated within the wider framework of the Lower Palaeolithic world [10, 11, 12, 13, 14]. Alongside bifaces, another noteworthy tool category in the Acheulian toolkit is the scraper. Scrapers are commonly defined as tools shaped on flakes with one edge modified to enable tasks other than straight-forward cutting. Scrapers appear in many Lower Palaeolithic sites and continue to be in the service of humans in later periods as well [15, 16, 17, 18, 19, 20]. While scrapers are usually interpreted as wood and hide working tools, recent evidence suggest their use in butchering activities as well [21, 22, 23, 24, 25, 26, 27, 28, 29]. While bifaces and scrapers were the foci of attention for generations of researchers, it is becoming clear now that small tools were also part of the Acheulian tool-kit, as evident by the production of miniscule items by means of recycling in several Lower Palaeolithic sites [30, 31, 32]. It is also of note here that during the Late Acheulian some significant technological innovation also took place, such as the appearance of prepared core technologies that developed further into the characteristic Levallois technology of the Middle Palaeolithic period [2, 33, 34].

Information regarding the function of Acheulian flint tools is still incomplete, particularly concerning bifaces. There are various hypotheses regarding the role of these enigmatic tools that varies from functional meaning, such as butchering and wood working [35, 36] to symbolic and social meaning [37, 38, 39].

Ungulate species are represented at Acheulian sites across the old world, and it is evident that a variety of mammalian taxa was preyed upon by Lower Palaeolithic hominins. Proboscideans, however, are ubiquitously found at many Palaeolithic sites, both open-air and caves, throughout a period of hundreds of thousands of years [2]. Elephants and mammoths were by far the largest terrestrial mammal available for Palaeolithic hominins, and these large animals represent a unique food package in terms of the composition of fat and meat [40]. The methods of obtaining meat by early humans is still under discussion. While some scholars still argue that in Palaeolithic times humans were scavengers, most researchers nowadays strongly advocate hunting as the principal method for obtaining calories, based on new results of sets of analyses of the archaeological record (e.g. [41, 42, 43, 44, 45, 46]). In this paper, however, we make no contribution regarding this hunting-scavenging debate but focus our attention on the use of Palaeolithic stone tools that follows the acquisition of the carcasses. Many Palaeolithic sites exhibit evidence of elephant and mammoths bones bearing cut marks indicating the removal of meat and percussion marks indicating the breaking bones for marrow extraction [47, 48, 49, 50]. In the Levant and elsewhere there are several Acheulian contexts characterized by the association of megafaunal remains and human made tools [40, 51, 52, 53], and in our opinion elephants had an important role in human diet and survival throughout the Palaeolithic [2, 40].

Data related to microwear studies focused on the investigation of Acheulean stone tools are few. However, evidence regarding the interpretation of the use of Acheulean tools and their possible relationship with processing big game comes from the studies of Keeley (1980) and Anzidei et al (2012) [54, 14]. In particular, in the work related to the British Lower Palaeolithic contexts of Clacton on Sea and Hoxne, where big game remains are present, Keeley (1980) describes several stone tools bearing both edge damage and microwear associated with meat cutting/butchering activities [54]. Moreover, at the site of La Polledrara di Cecanibbio, characterized by the presence of hundreds of elephant remains, Anzidei and colleagues (2012) describe numerous lithic implements exhibiting use wear associable to butchering activities and hide processing [14]. Unfortunately, the direct relation between the tools and the elephant bones remains to be demonstrated, as neither at Claton on Sea, Hoxne and La Polledrara no cut marks have yet been found on the tools.

Residue analysis has been performed on stone tools from several prehistoric contexts in order to understand the use of the tools and the materials worked [24, 35, 55, 56, 57, 58, 59, 60, 61]. In order to determine the molecular composition of organic residues of animal or vegetable origin gas chromatography –mass spectrometry (GC-MS) is employed. This analysis requires the extraction of small amount of the residues analyzed using, for example, the Fatty Acid Methyl Ester Technique [62]. The Fourier Transform Infra Red (FTIR) analysis is often combined with more invasive techniques for the identification of archaeological residues such as resins [63], pigments [64] or organic materials [65, 66]. More recently, the micro Fourier Transform Infra Red (FTIR) technique which is non invasive and does not requires any chemical or mechanical pre-treatment of the samples has been successfully employed in order to identify residues on lithic items [58, 67].

However, only few such studies have been conducted on tools from very early archaeological contexts, such as the Lower Palaeolithic Acheulian. Herrygers (2002) was able to detect wood residue on Oldowan tools from Koobi Fora, this results support the conclusion drawn from use-wear analysis carried out by Keeley and Toth (1981) [57, 68]. Another example is given by Dominguez-Rodrigo et al (2001) who identified phytoliths on several handaxes from the Acheulian site of Peninj [35]. Hardy and Moncel (2011) identified several plant and animal residues on stone tools related to the Neanderthal Middle Palaeolithic occupation excavated at the site of Payre (France) [56]. Only few works (e.g. [58, 60, 61, 69, 70]) included a combination of both use-wear and residues analyses.

The goal of our research is to study in detail flint tools directly associated with butchered game remains from a well-preserved archaeological context at Late Acheulian Revadim. We work under the assumption that the extraordinary preservation circumstances in this context will allow the application of use-wear analysis and FTIR analysis for the understanding of the function of Palaeolithic stone tools in the Acheulian. Our research question is whether the application of residue and use wear analyses on well preserved Acheulian stone tools could shed new light on the function of the tools and the material processed in this context. Moreover, we would like to envisage the implications of such analyses on the broader understanding of human uses of both flint and animal resources in the Palaeolithic. This paper will present the results originating from the application of an integrated use-wear and residues approach, finalized to the interpretation of the use of a biface and a scraper recovered from a specific archaeological context at the Acheulian site of Revadim, Israel and associated with prey mammal bones in general and butchered elephant remains in particular.

### Site description

The Late Acheulian site Revadim is located on the southern coastal plain of Israel. The site is at least ca. 500–300 thousand years old, as indicated by minimum ages retrieved by paleomagnetism analysis and uranium series dates performed on carbonate coating on flint items [48, 71, 72]. Archaeological excavations at the site of Revadim were conducted by one of us (OM) under the permission of the Israel Antiquities Authority (Permit A-41091). Four excavation seasons were conducted on the site. The last excavation season was a salvage excavation carried out in the summer of 2004 and focusing on two main areas, B and C. In area C five stratigraphic layers were identified: C1-C5, while in Area B two layers, B1 and B2, were identified.

Area B spread on 94 m² and the excavated sediments volume is 50.17 m³. The lithic assemblage includes 27,591 flint items, with an average density of 550 items per m^3^. Debitage, cores and shaped items comprise 17.14% of the assemblage in the area (Table 1), including 7% (n = 1988) shaped items ("tools").

**Table 1:** Flint assemblage of Area B, Revadim.

Area B assemblage	Number	Percentage of débitage and shaped items general	Percentage of total assemblage general
Primary Elements Flake	174	3.7	0.6
Primary Elements Blade	4	0.1	0.0
Flakes	763	16.2	2.8
Broken flakes	1222	25.8	4.4
Blades	18	0.4	0.1
Cores	213	4.5	0.8
Core Trimming Elements (CTE)	179	3.8	0.6
Cores on flake	78	1.6	0.3
Tools	1988	42.0	7.2
Special Spalls	89	1.9	0.3
**Sum**	**4728**	**100**	**17.1**
Micro flakes	1308		4.7
Chips	20358		73.8
Chunks	928		3.4
Flaked pebbles	178		0.6
Raw materials	91		0.3
**Total**	**27591**		**100**

Thousands of animal bones were found in each layer of the site. The faunal assemblage include *Palaeloxodon antiquus*, *Bos primigenius*, *Capra cf*. *aegagrus*, *Gazella gazella*, *Cervus elaphus*, *Dama cf*. *mesopotamica*, *Equus sp*. *among others* [48].

Straight tusk elephant, *Palaeloxodon antiquus*, is the most dominant species in the faunal assemblages of Revadim, with 155 bones identified [48] and it's remains were found in all archaeological layers.

Some of the elephant bones were found in discrete localities identified in Area B representing, most probably, specific activity areas that were relatively well-preserved. In some of these localities the density of finds is higher than in the surrounding areas, and in other cases the localities have specific characteristics, such as special sediment or special items articulation. For example Locality 20 (southern part of Area B) have a density of 4462.5 lithic items per m³, while the findings density in the surrounding area is 1,554 items per m^3^. Another example is Locality 22 which is located in the northern part of Area B, and it is characterized by special items articulation as 3 bifacial tools were found close to a raw material pebble [73]. It's worth noting that part of this localities are the result of special post depositional activities such as localities 2–3 (see [48] for details).

This paper will present data from Locality 21, a well-preserved fast-palimpsest that exhibits elephant remains in association with flint tools.

**Fig 1:**
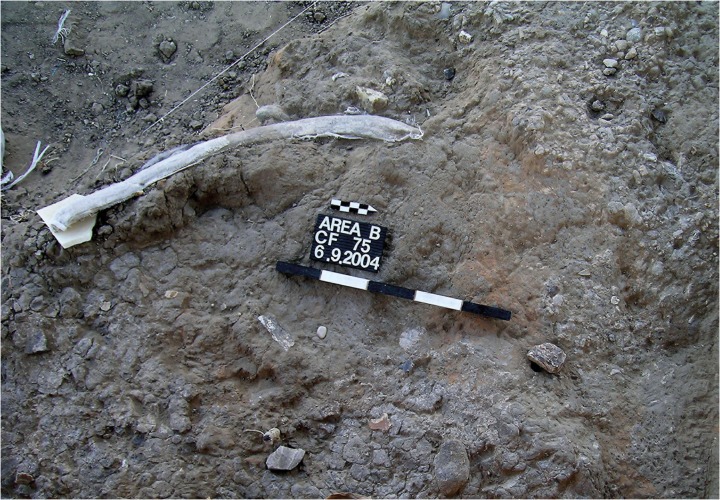
Locality 21—elephant rib with cut marks in association with flint items (including handaxe).

Locality 21 is a concentration of finds that was defined during the excavation of Layer B2 in the central part of Area B. This locality is characterized by distinctive sediment, different than its surroundings, and probably represents a genuine activity area that was covered rather quickly after it was deposited (fast palimpsest). Locality 21 is one of three find concentrations in Area B where elephant remains are found alongside flint tools (Fig. 1 and see [48, 73] for details).

Locality 21 includes 984 flint items (see details in Table 2). The density of lithic items (547 per m^3^) is slightly higher than the average density of findings in the entire central area in this layer (433.6 items per m^3^) and the excavated volume of this locality is 1.8 m^3^.

**Table 2:** Flint assemblage of Locality 21.

Locality 21 (Central part of Area B), Layer B2	Number	Percentage of débitage and shaped items Locality 21	Percentage of total assemblage Locality 21
Primary Elements Flake	7	3	1
Primary Elements Blade	2	1	0
Flakes	29	11	3
Broken flakes	64	25	7
Blades	1	0	0
Cores	33	13	3
Core Trimming Elements (CTE)	8	3	1
Cores on flake	4	2	0
Tools	108	42	11
Special Spalls	3	1	0
**Sum**	**259**	**100**	**26**
Micro flakes	32		3
Chips	612		62
Chunks	52		5
Flaked pebbles	10		1
Raw materials	19		2
**Total**	**984**		**100**

Flint raw material items and cores constitute a significant part of locality 21 lithic assemblage. The ratio between blanks (flakes and flake tools) and cores is 6:1, indicating non-intensive lithic production in this locality compared to other areas within the site, e.g. in layer B2 this ratio is 18:1 (see [73] for details).

Bifaces are rather frequent in locality 21 assemblage (n = 15) and comprise 14% of the shaped items ("tools"). In addition, two biface tips were recovered as well suggesting that biface use and/or maintenance have taken place within locality 21 (Table 3).

**Table 3:** Shaped items (tools) of Locality 21.

Shaped items (tools)—Locality 21	Number	Percentage of total tools assemblage
Retouched flakes	34	31
Retouched primary elements flake	1	1
Retouched broken flakes	10	9
Retouched micro flakes	7	6
Retouched blades	0	0
Shaped special spalls	0	0
Sidescrapers	4	4
Endscrapers	1	1
Awl/Borers	1	1
Truncations	2	2
Notches	15	14
Denticulates	2	2
Burins	1	1
Varia	15	14
Bifaces	15	14
Choppers	0	0
**Total**	**108**	**100**

Notable faunal remains that were found in this Locality are an ulna shaft of *Dama*, *Equus* tooth, and elephant remains (two elephant ribs, vertebrae plate, and seven teeth fragments). On one of the elephant ribs cut marks were identified [48] (6, Table 3).

## Materials and Methods

### Samples

Three flint items [a flake (Sq. CG 76), a scraper (item no. 103070) and a biface (item no. 103070), housed at the institute of archaeology, Tel-Aviv University, Israel] originating from activity area Locality 21 were selected to undergo a Fourier Transform InfraRed (FTIR) analysis in order to underline the presence of preserved residues. The scraper and biface were selected during use-wear analyses, where all bifaces (n = 15) and all scrapers (n = 4) of Locality 21 were examined. Traces were found on 4 bifaces and one scraper, but only two out of these five tools presented both types of traces—edge removals and polish. For this reason and for general good level of preservation of these two items they were selected for FTIR analysis as carries potential residue.

One flake was chosen during use-wear analyses of all flakes (n = 29) from Locality 21, snce red dots where recognized on this flake through the metallographic microscope (technical details will be described in this section).

### Use-wear analysis

The method of use-wear analysis was developed in the middle of the 20^th^ century by S.A. Semenov [74, 75]. Some researchers influenced by Semenov's research method, including Tringham and Keeley who contributed to its development [76, 77].

Tringham and Odell used a low-powered approach, concentrated on micro removals on the working edge. Keeley used a more powerful microscope and he examined polish, a method named high-power approach [76, 78, 79].

In this work use-wear analysis was conducted by using both low and high power magnification approaches [75]. Low-power approach is generally performed utilizing a stereo microscope, and focused on the identification and description of edge damage (e.g. fractures and edge rounding), while high-power approach focuses on the investigation of polishes, striae and abrasions, visible at higher (100x and more) magnifications. [76, 78, 79].

The items analyzed were cleaned only in H2O with soap before use-wear analyses. The analysis was conducted with two different types of microscopes: an Olympus SZ-PT stereo-microscope with oculars magnification 10x and a Nikon Optiphot metallographic microscope with oculars magnification 10x and objectives 10x and 20x; both equipment were associated with a reflect light system.

### FTIR spectroscopy

The Fourier Transform InfraRed (FTIR) spectra of the stone tools were collected with a Bruker Optic Alpha-R portable interferometer with an external reflectance head covering a circular area of about 5 mm in diameter. The samples were placed directly in front of the objective, without preliminary treatments, and spots were selected for analysis. The analysis does not require preliminary treatment of the samples. The investigated spectral range was 7500-375 cm^−1^ with a resolution of 4 cm^−1^ cumulating 250 scans or more.

Figures reported in the following, however, show only the spectral range where infrared active absorption bands due to fundamental modes were observed (4000–375 cm^−1^) since no overtones or combination bands expected at higher frequencies were detected. Internal point and edges of the tools were examined in the same experimental conditions.

## Results

Here we summaries the results of use-wear and residue analyses conducted on the three flint items from Locality 21 found in association with elephant butchered bones. Each item will be presented separately, where use-wear analysis will be shown first and the residue analysis will follow.

### Flint Flake (Sq. CG 76)

#### Use-wear

**Fig 2:**
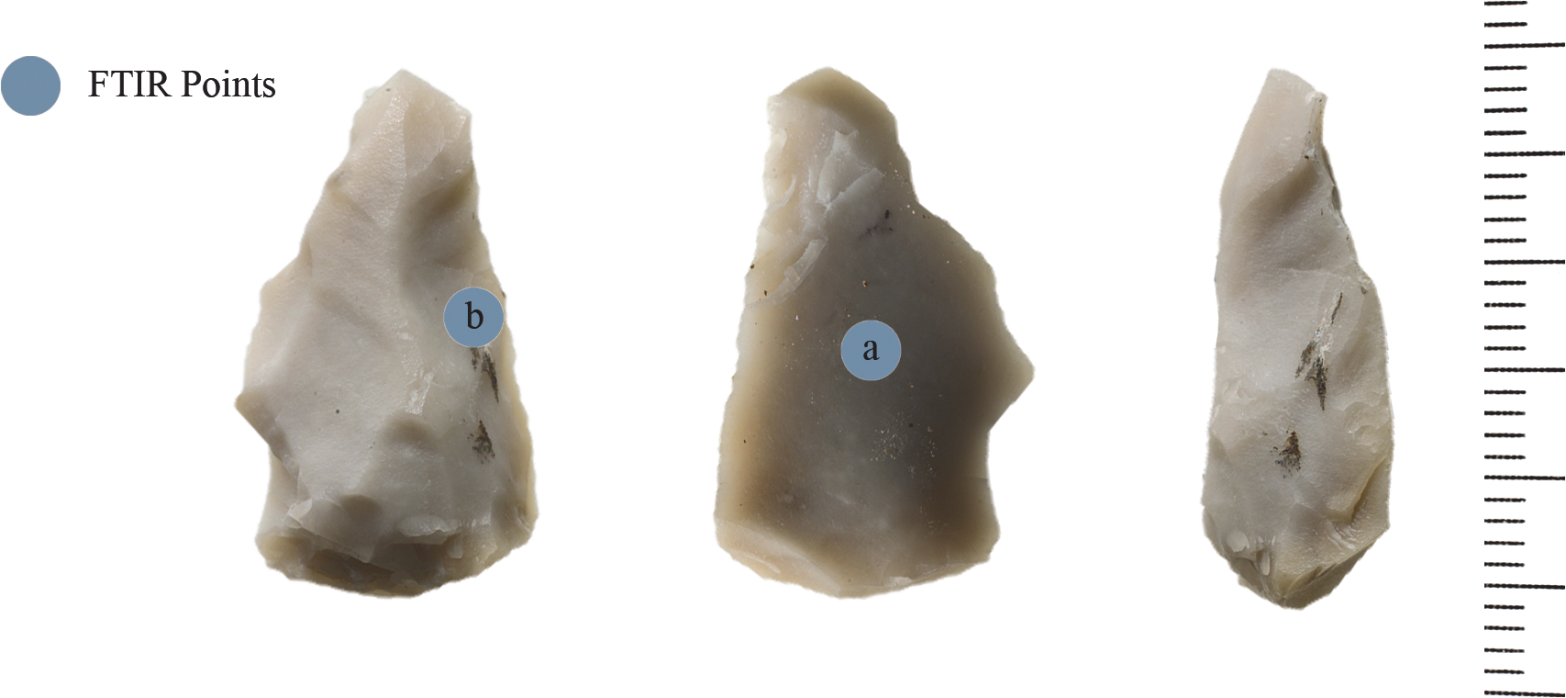
Flake CG76.

On a flint flake made from homogeneous raw material from square CG76 (Locality 21) no diagnostic wear traces were recognized (Fig. 2). However during use-wear analysis the presence of red dots was identified. The flake is 25 mm in length, 14 mm wide and 6 mm thick.

#### FTIR analysis

**Fig 3:**
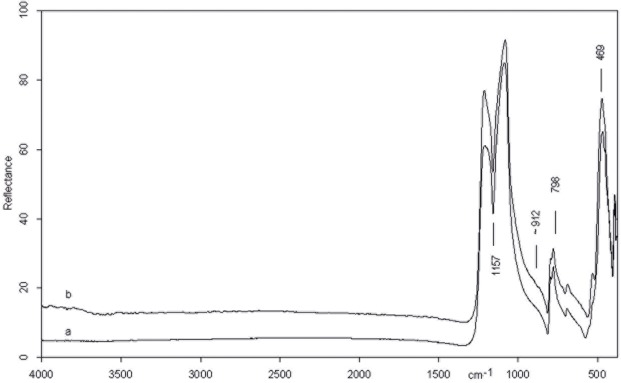
Micro FTIR spectrum of CG 76 flake: inner point (a) showing the fundamental mode of pure silica and point on the edge on dorsal face (b) with bone microresidue (see text).

The spectrum of the central point of the CG-76 item shows the absorption bands belonging to pure silica (Fig. 3A). The very intense peak at 1157 cm^−1^, in fact, is assigned to Si-O stretching mode and less intense peaks at 798 and 469 cm^−1.^ Are attributed to O-Si-O bending modes and to O-Si-O or O-Si-Al bending respectively [80, 81]. All peaks show an up down reversal that is the restrahlung effect which, at present, is not completely explained [82]. The spectrum of the edge on dorsal face (Fig. 3B), instead, shows a shoulder on the low frequency side of the Si-O stretching mode (∼912 cm^−1^) which suggests the presence of bone microresidues since it is attributable to the PO_3_^=^ stretching mode of calcium phosphate (apatite) which constitutes the bone mineral component [83].

### Biface (item no. 103070)

**Fig 4:**
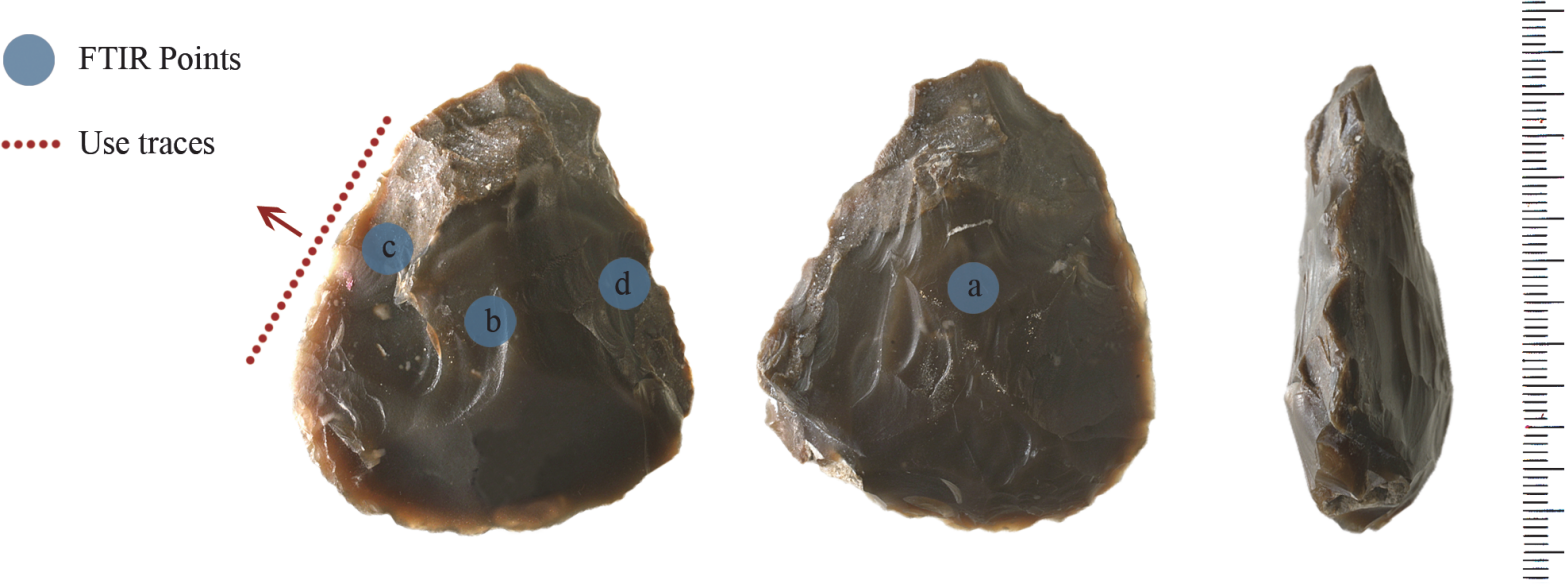
Biface 10307.

A complete small biface, most probably made on flake, was analyzed (Fig. 4). The flint raw material used is homogeneous with some internal impurities. The biface is 56 mm in length, 48 mm wide and 16 mm thick. It bears no cortex.

The dorsal face of the biface is intensively knapped by bifacial flaking, while part of the ventral face remained unshaped.

#### Use-wear

The biface is characterized by a medium degree of edge rounding, along with the presence of edge damage scars bearing cone initiations and hinge like terminations. These scars are characterized by a perpendicular orientation to the edge of the biface while their distribution over the edges is variable.

**Fig 5:**
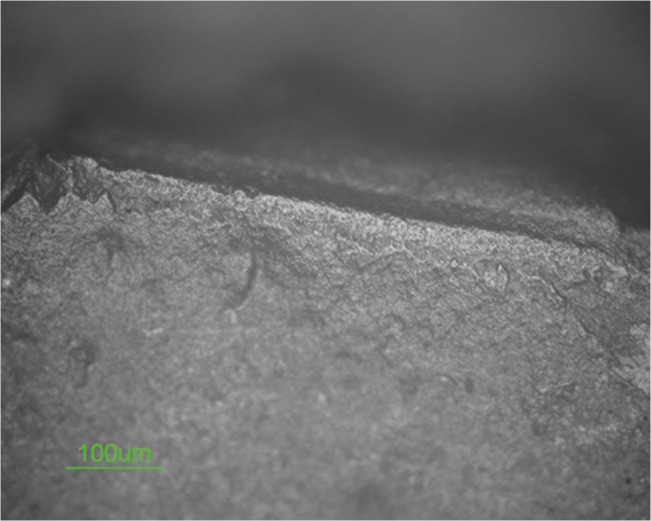
Polish on biface 10307.

Micro wear was detected as well. Rough to smooth polishes are visible on the bifacially-shaped edges of the biface. Their extension is limited to the outer portion of the edge and its direction is perpendicular. The identification of both edge damage and micro wear features enable to define the utilization of this biface in processing medium hard material, probably hide, throughout transversal movements, which may be related to scraping (Fig. 5).

#### FTIR analysis

**Fig 6:**
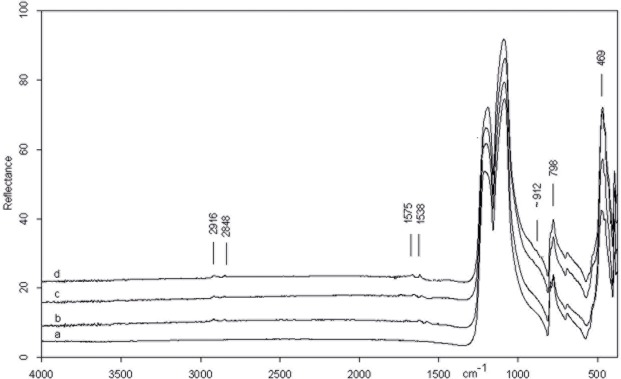
Micro FTIR spectrum of biface 10307: (a) pure silica, (b) inner point, (c) left edge, (d) right edge. Microresidues of *adipocere* are detected on points b, c and d. On the right point (d) also a micro remain of bone is detected (see text).

Fig. 6 shows the micro spectra of the three points examined on the biface, as shown in Fig. 4 (An inner point 6b, a left point and a right point 6c and 6d). For the sake of comparison the spectrum of pure silica is also reported (Fig. 6B).

The spectra of the central point and edges of the item show very weak bands at 1575\1538 cm^−1^ and 2916 and 2848. cm^−1^. The former doublet is confidently assigned to the C-O stretching of calcium salt carboxylate of saturated acids constituting the *adipocere*, a wax-like organic substance formed by the anaerobic bacterial hydrolysis of fat tissues [84, 85]. The presence of organic residue is supported by the presence of C-H stretching modes at 2916 and 2848 cm^−1^.

In addition, in the spectrum of the right edge, the shoulder at ∼ 912 cm^−1^ appears indicating, as previously discussed, the existence of bone micro residues.

### Scraper (item no. 10982)

**Fig 7:**
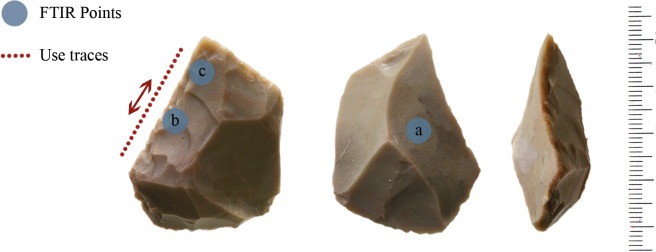
Scraper 10982.

A convergent scraper with steep retouch on its dorsal face was analyzed (Fig. 7). It is made on homogeneous flint without impurities. The scraper is 44 mm in length, 36 mm wide and 20 mm thick.

It is important to note that this tool is rather unique first of all because of the high quality raw material used, which is not typical for Revadim site. Furthermore, convergent scrapers are not common in Acheulian lithic assemblages as well. It seems that this tool can indicate number of use cycles which include refinement and repair of the working edges. It is possible to assume that this scraper is more similar to scrapers characteristics of the later periods in the Levant, such as the succeeding Acheulo-Yabrudian cultural complex [2].

#### Use-wear

**Fig 8:**
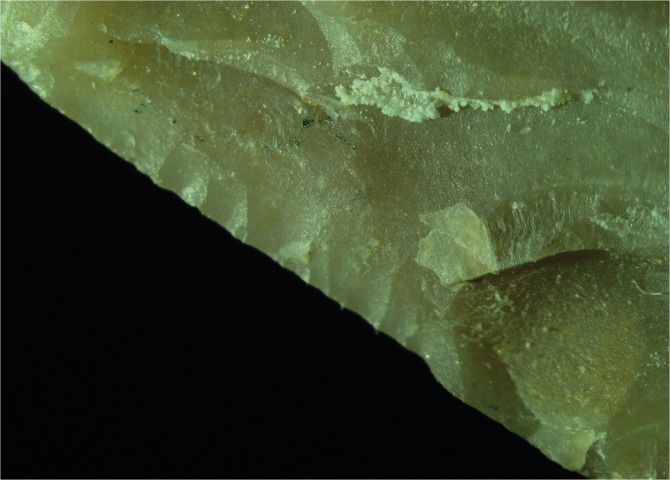
Edge damage on scraper 10982.

The scraper is characterized by both edge damage and micro wear. Scars characterized by feather terminations are present on the tool’s edge dorsal surface. These scars exhibit a diagonal unidirectional orientation and a close regular distribution. Edge rounding is present as well at a low degree (Fig. 8).

**Fig 9:**
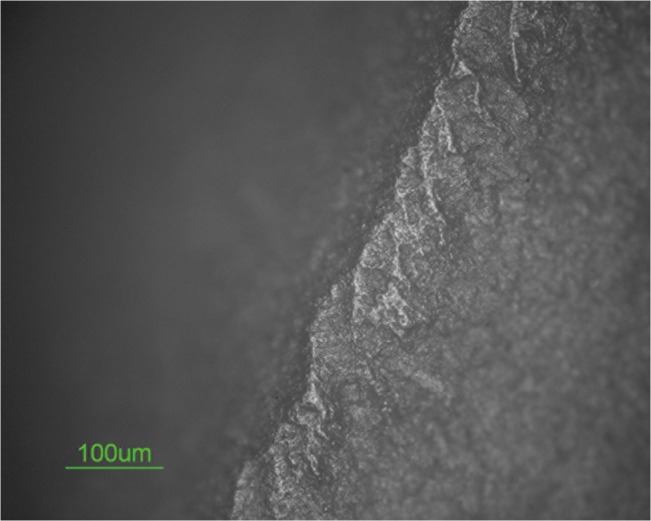
Polish on scraper 10982.

Micro wear is represented by two different types of polishes featuring a rough texture, one is characterized by a domed like topography, while the other is not as developed to allow a description of its topography. Their extension is limited to the outer edge of the tool and its direction is diagonal unidirectional. The features exhibited both by edge damage and micro wear indicate that the scraper was used to process soft and medium materials performing longitudinal motions related to a probable cutting activity. The two different types of polish present on the scraper’s edge may indicate the use of the tool to process some sort of animal tissues and wood. This interpretation is given by the domed topography of one of the polishes developed on the tool (Fig. 9).

#### FTIR analysis

**Fig 10:**
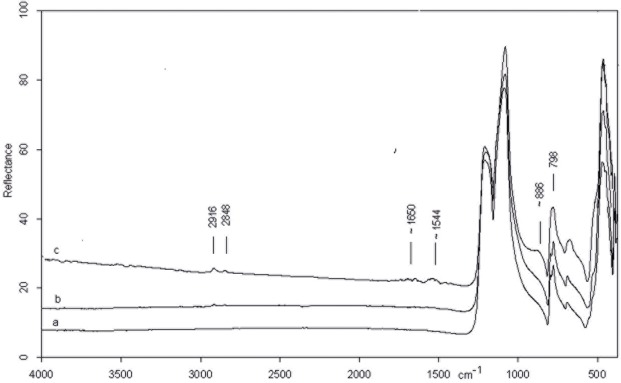
Micro FTIR spectrum of scraper 10982: (a) pure silica, (b) edge point, (c) inner point. Microresidues of organic material probably of vegetal origin are detected on the inner point (see text).

Fig. 10 compares the micro FTIR spectra of the inner point and the point on the edge in dorsal face of the scraper, as shown in Fig. 7.

The spectrum of the inner point (Fig. 10A) presents weak broad features at about 1660 and 1544 cm-1 and a shoulder at the low frequency side of the Si-O intense peak (∼886 cm-1). The additional presence of weak bands at 2848 and 2912 cm-1 belonging to O-H stretching suggests the presence of an organic residue probably of vegetable nature [86]. A more precise identification is however not possible.

On the dorsal edge the afore mentioned doublet around 1500 cm^−1^ and the shoulder at ∼912 cm-1 indicates that the scraper had a prolonged contact with animal tissues (Fig. 10B-C).

## Discussion and Conclusion

The combination of use wear and residues analyses to investigate the use of Palaeolithic tools is not widely used in archaeological research. Moreover, there are only few examples of the preservation of organic residues on artifacts originating from Lower Palaeolithic / Early Stone Age contexts [35, 56, 57, 68], and thus our finding represents an unique evidence.

Here we presented the data obtained through the analysis of both use wear and residues found on two flint tools, a biface and a scraper, from the late Acheulian site of Revadim, Israel. The implements (a flake, a biface and a scraper) were analyzed in order to investigate and define possible function of these specific types of artifacts.

The flake was selected because of its fresh surface and for the presence of red points on its' surface which may indicate some sort of residue. Unfortunately no use-traces or identifiable residue was found. Bone micro residue was recognized on this tool, and also on the biface described below; probably indicating the presence of bones micro-particles in the sediment of the site, as no use wear related to bone or hard animal materials was identified on the tools.

The biface and scraper show well preserved and clear use-wear traces, including both edge removals and polishes. The biface use traces indicate the processing of medium-hard material throughout transversal motions, related probably to hide scraping. In the case of the scraper, the traces shows attributes associated with soft or medium material processed throughout longitudinal motion. The possible interpretation is cutting of animal tissues. Another kind of polish, that could be associated with wood, was also detected.

Both tools shows residue of *adipocere* (animal fat) which can be related to butchering (de-skinning) activity or hide working. On the scraper, both tissues and vegetable materials residues were found.

In this work we would like to stress the importance of the data obtained by the application of an approach combining both use wear and residues analyses. The simultaneous application of the two techniques allowed us to obtain more detailed evidence regarding the use of the two most characteristics shaped stone tools of the Lower Palaeolithic period, bifaces and scrapers.

Our results allow us to speculate about the possible use of these tools in processing animal carcasses. Elephants, being the most common animal within the faunal assemblage on site, and the presence of cut marks on an elephant rib found in Locality 21, in particular, support our suggestion that these stone tools might have been used on elephant carcasses (and other game as well).

Furthermore, such direct data related to the use of Acheulian tools is overall not common, mainly due to the level of preservation of the materials coming from such old contexts. The data here presented provide clear and direct evidence for possible way of use of stone tools at Revadim, and enables a better understanding of the behaviors related to the early human groups occupying the Levant during the Lower Palaeolithic period.

## Supporting Information

S1 Fig Location map of Revadim Quarry. (TIF)

S2 Fig Areas of excavation. (TIF)

S3 Fig Locality 21—general view. (TIF)

S1 Table Shaped items (tools) of Area B. (TIF)

S2 Table Flint assemblage of Layer B2. (TIF)

S3 Table Shaped items (tools) of Layer B2. (TIF)

S4 Table Table of edge damage attributes for both items (handaxe 10307 and scraper 10982). (TIF)

S5 Table Table of micro wear attributes for both items (handaxe 10307 and scraper 10982). (TIF)
