# Supplementary figures and images for: Fat Residue and Use-Wear Found on Acheulian Biface and Scraper Associated with Butchered Elephant Remains at the Site of Revadim, Israel
Source: PLoS One. 2015 Mar 18;10(3):e0118572. doi: 10.1371/journal.pone.0118572 (PMC4365021; doi:10.1371/journal.pone.0118572)

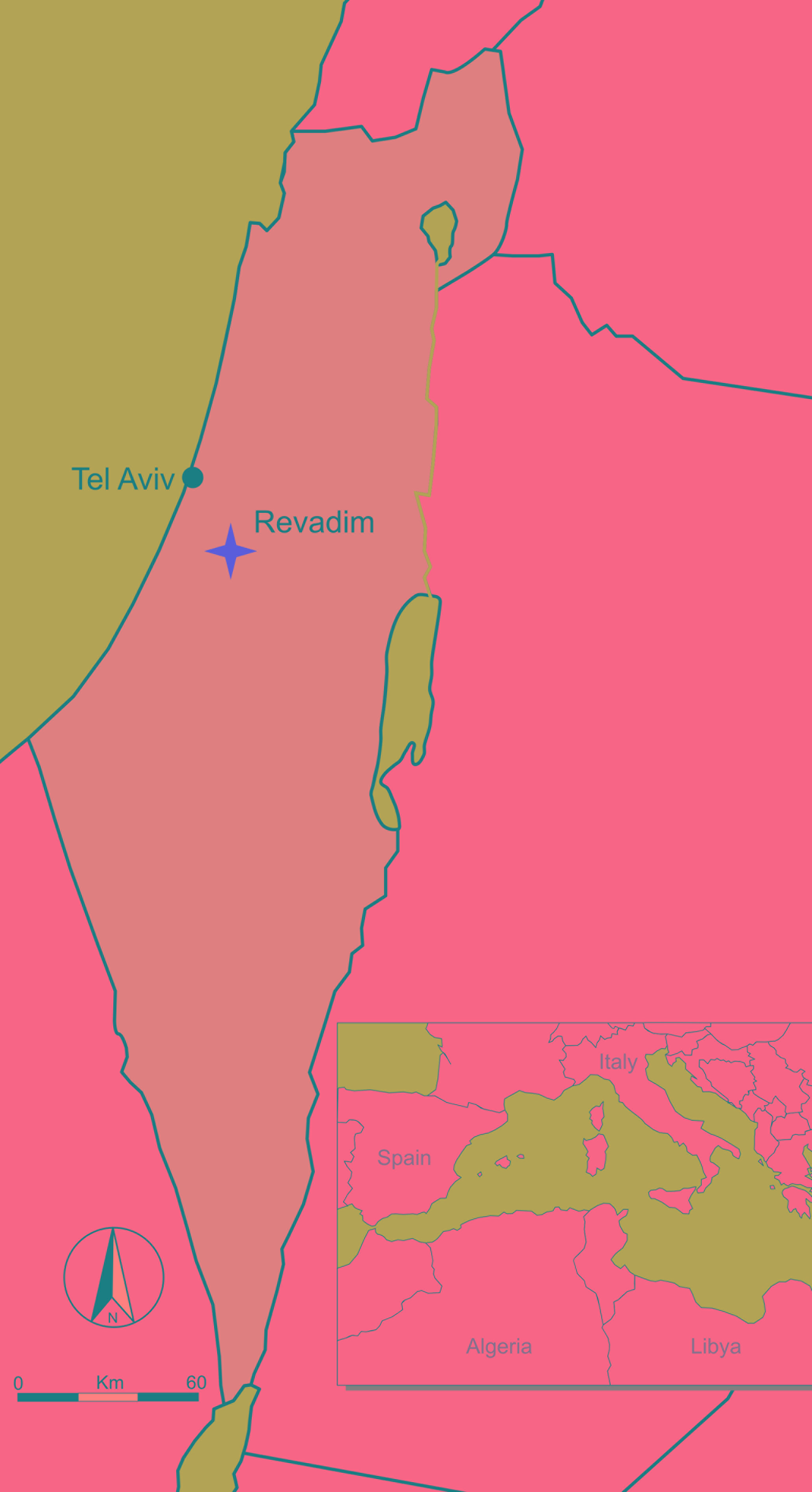

Supplement: S1 Fig — (TIF) [file pone.0118572.s001.tif]

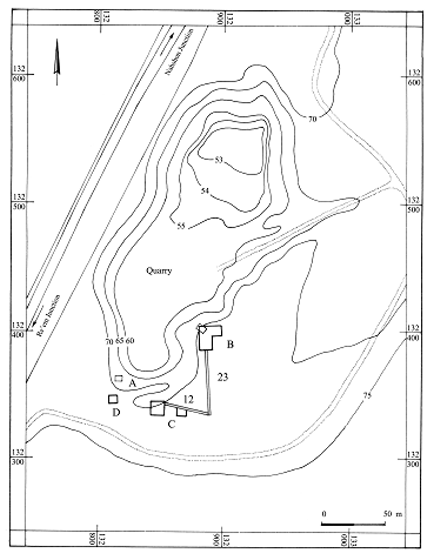

Supplement: S2 Fig — (TIF) [file pone.0118572.s002.tif]

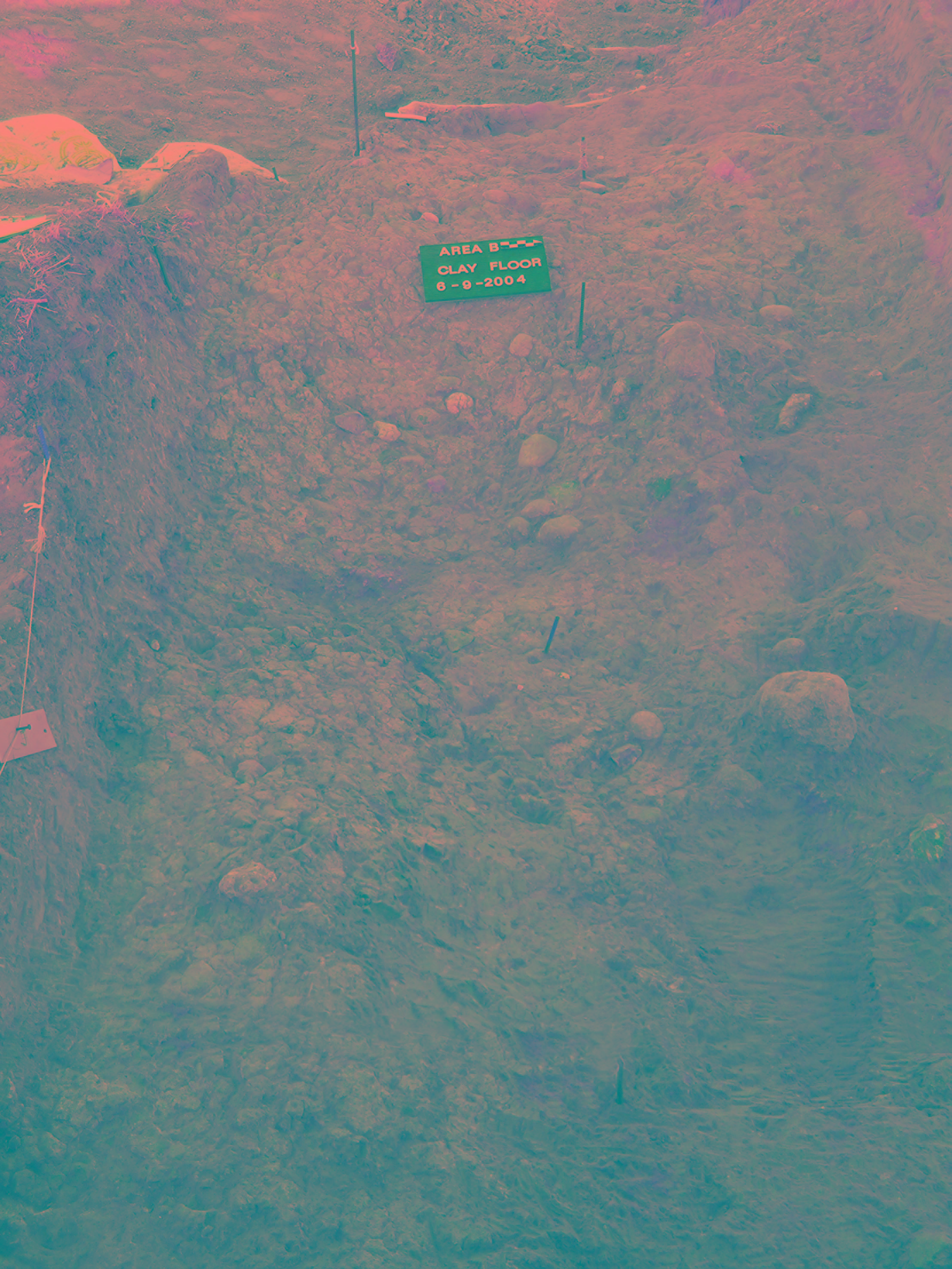

Supplement: S3 Fig — (TIF) [file pone.0118572.s003.tif]

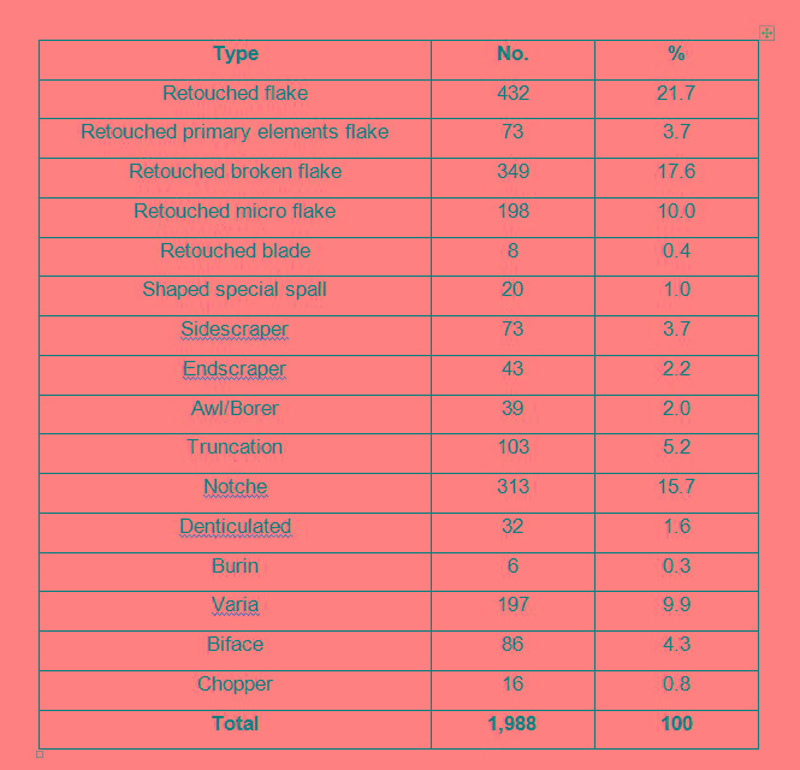

Supplement: S1 Table — (TIF) [file pone.0118572.s004.tif]

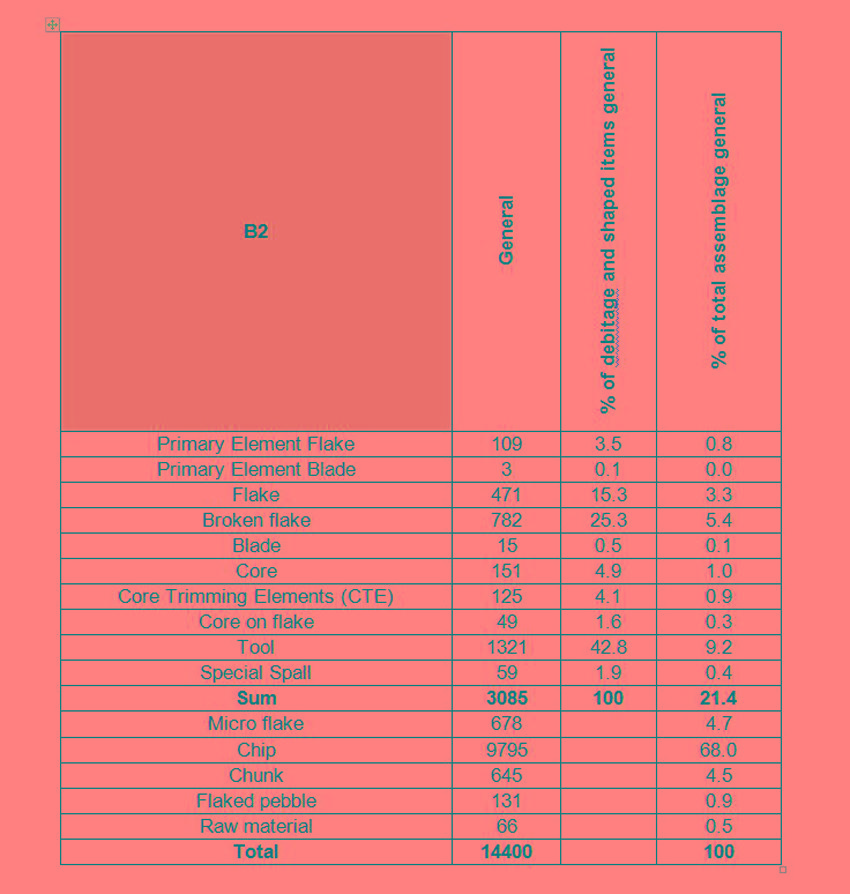

Supplement: S2 Table — (TIF) [file pone.0118572.s005.tif]

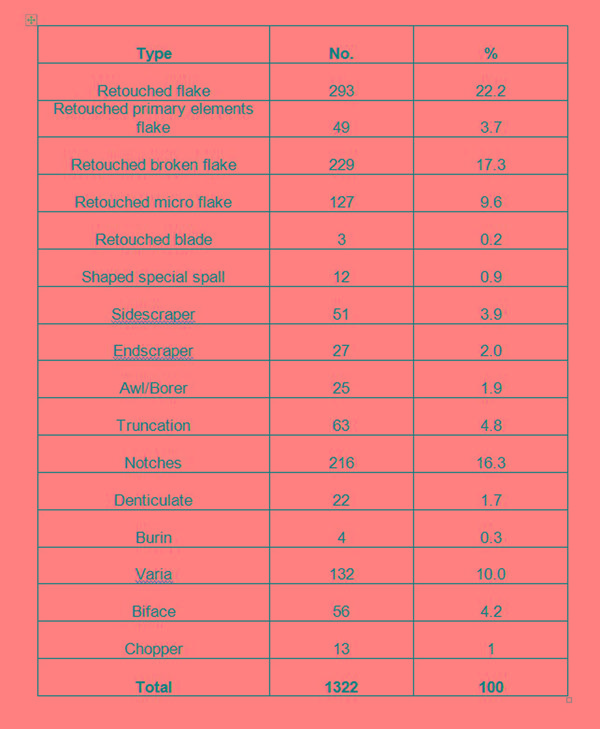

Supplement: S3 Table — (TIF) [file pone.0118572.s006.tif]

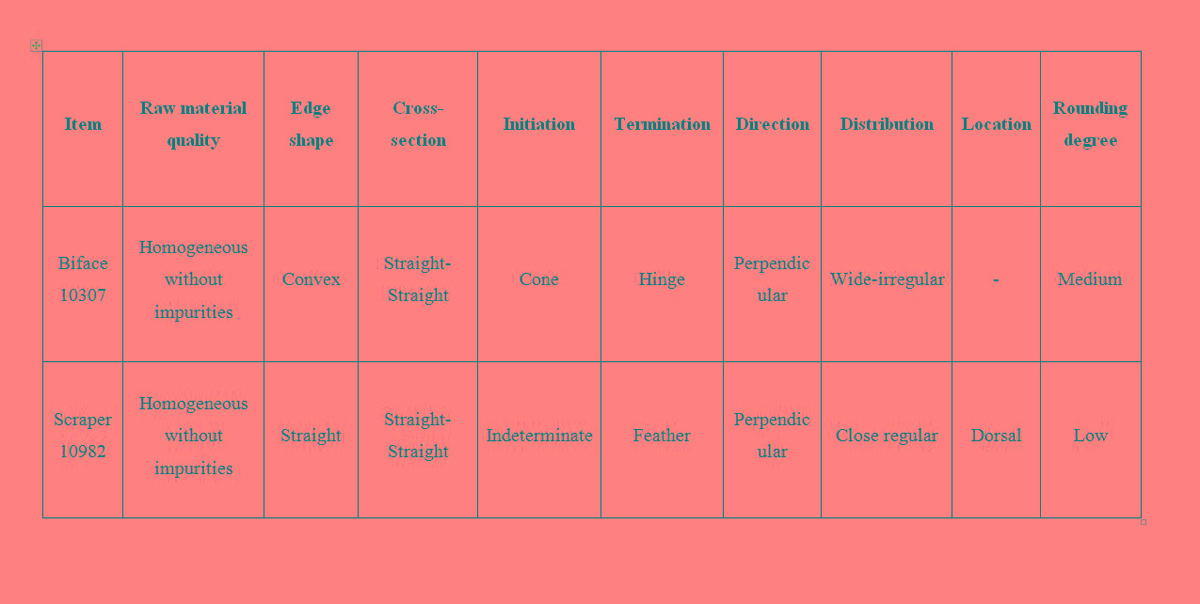

Supplement: S4 Table — (TIF) [file pone.0118572.s007.tif]

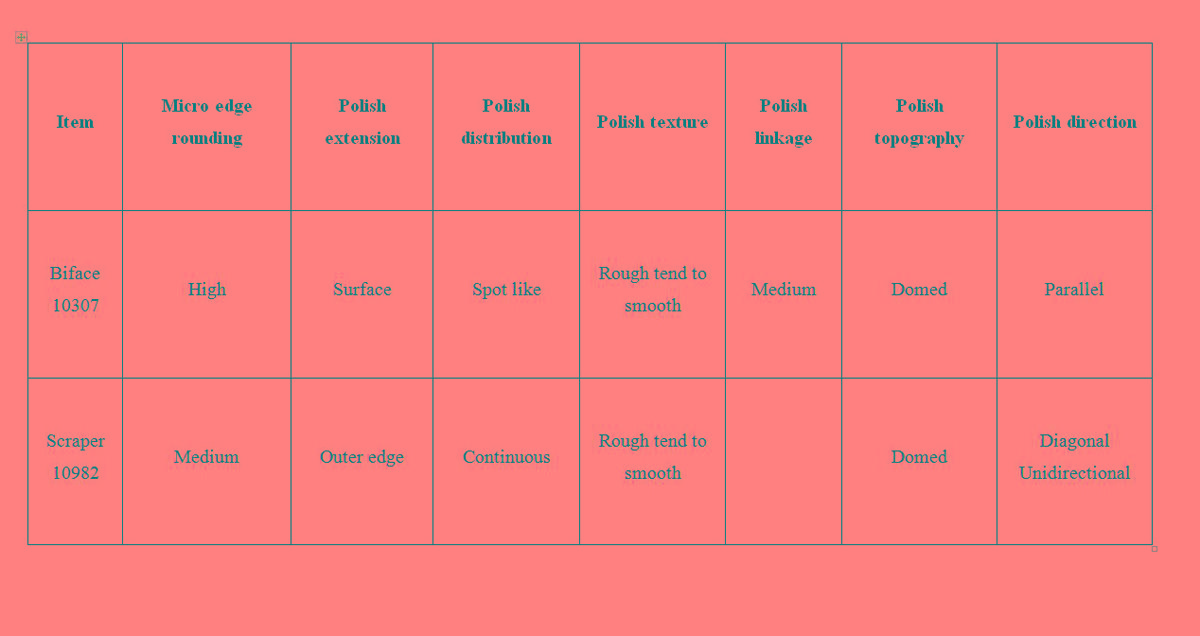

Supplement: S5 Table — (TIF) [file pone.0118572.s008.tif]
